# Quality and Perception of Attention-Deficit/Hyperactivity Disorder Content on TikTok: Cross-Sectional Study

**DOI:** 10.2196/75973

**Published:** 2025-11-13

**Authors:** Katharina Sieferle, Tiziana Guidi, Florence Dorr, Eva Maria Bitzer

**Affiliations:** 1 Public Health and Health Education, University of Education Freiburg, Kunzenweg 21, Freiburg im Breisgau, 79117, Germany, 49 761 682755

**Keywords:** social media, health information, misinformation, attention-deficit/hyperactivity disorder, ADHD, TikTok

## Abstract

**Background:**

Social media platforms are increasingly used for both sharing and seeking health-related information online. TikTok has become one of the most widely used social networking platforms. One health-related topic trending on TikTok recently is attention-deficit/hyperactivity disorder (ADHD). However, the accuracy of health-related information on TikTok remains a significant concern. Misleading information about ADHD on TikTok can increase stigmatization and lead to false “self-diagnosis,” pathologizing of normal behavior, and overuse of care.

**Objective:**

This study aims to investigate the quality and usefulness of popular TikTok videos about ADHD and to explore how this content is perceived by the viewers based on an in-depth analysis of the video comments.

**Methods:**

We scraped data from the 125 most liked ADHD-related TikTok videos uploaded between July 2021 and November 2023 using a commercial scraping software. We categorized videos based on the usefulness of their content as “misleading,” “personal experience,” or “useful” and used the Patient Education Materials Assessment Tool for Audiovisual Materials to evaluate the video quality regarding understandability and actionability. By purposive sampling, we selected 6 videos and analyzed the content of 100 randomly selected user comments per video to understand the extent of self-identification with ADHD behavior among the viewers. All qualitative analyses were carried out independently by at least 2 authors; the disagreement was resolved by discussion. Using SPSS (version 27; IBM Corp), we calculated the interrater reliability between the raters and the descriptive statistics for video and creator characteristics. We used one-way ANOVA to compare the usefulness of the videos.

**Results:**

We assessed 50.4% (63/125) of the videos as misleading, 30.4% (38/125) as personal experience, and 19.2% (24/125) as useful. The Patient Education Materials Assessment Tool for Audiovisual Materials scores for all videos for understandability and actionability are 79.5% and 5.1%, respectively. With a score of 92.3%, useful videos scored significantly higher for understandability than misleading and personal experience videos (*P*<.001). For actionability, there was no statistically significant difference depending on the videos’ usefulness (*P*=.415). Viewers resonated with the ADHD-related behaviors depicted in the videos in 220 out of 600 (36.7%) of the comments and with ADHD in 32 out of 600 (5.3%) of the comments. Self-attribution of behavioral patterns varied significantly, depending on the usefulness of the videos, with personal experience videos showing the most comments on self-attribution of behavioral patterns (102/600, 17% of comments; *P*<.001). For self-attribution of ADHD, we found no significant difference depending on the usefulness of the videos (*P*=.359).

**Conclusions:**

The high number of misleading videos on ADHD on TikTok and the high percentage of users who self-identify with the symptoms and behaviors presented in these videos can potentially increase misdiagnosis. This highlights the need to critically evaluate health information on social media and for health care professionals to address misconceptions arising from these platforms.

## Introduction

Social media use is continuously rising, and by early 2024, the number of social media users had reached over 5 billion worldwide, with users spending on average approximately 2.5 hours on social media every day [1]. Besides the popular platforms such as Facebook, YouTube, WhatsApp, and Instagram, the relatively new social media platform TikTok has seen a rise in popularity and users in the last few years. In 2024, it ranked fifth in the most popular social networks worldwide, with approximately 1.6 billion users per month [1] and was the most downloaded social media app in recent years [2]. TikTok differs from other social media or social networking platforms in the sense that users can publish posts only in the form of pictures or short videos, and that it is mainly used for entertainment. In addition, video descriptions are rather short. While it is possible to search for specific videos, hashtags, or creators, TikTok’s main feature is its algorithm, which creates a so-called “For You Page” for the user, by making personalized video recommendations based on analysis of previous engagement with videos [2,3]. Originally, TikTok primarily targeted Gen Z but expanded and diversified its user base during the COVID-19 pandemic [3]. Nonetheless, according to data from 2023, 71% of its user base is aged 18 to 34 years and therefore younger than the user base of many other popular social media platforms [4-6].

TikTok is mainly used for networking and entertainment, but increasingly also for both sharing and searching for health-related information online [7-9]. Social media offers the possibility to connect with other patients or a community with similar health conditions, seek emotional support, share personal experiences concerning diseases or medical treatments, and obtain information in an easily understandable format, such as pictures and short videos [7,10].

On TikTok, the popularity of health-related content began with the COVID-19 pandemic, but now there is information on a variety of health topics to be found, including diabetes, dental care, cancer, and mental health issues such as depression, autism spectrum disorder, and attention-deficit/hyperactivity disorder (ADHD) [8,9]. Especially the topic of ADHD has gained popularity on TikTok in recent years. The growing interest is evident from the increase in content under the hashtag “#adhd” in 2023 from 2 million to over 3 million videos within only 12 months [11].

Many TikTok videos about ADHD feature creators sharing their personal experience of the disorder. This can have many benefits for people with ADHD, as it can raise awareness about the disorder, help people understand symptoms and behavior typical for ADHD, and reduce stigmatization [12]. In addition, getting more information on the disorder and seeing and hearing people talk about their experiences with it can lower the threshold for seeking a diagnosis and potential treatment for individuals suffering from ADHD. A diagnosis is important to receive appropriate treatment and to target symptoms [13].

However, health information on social media and particularly TikTok should be viewed with caution, as several studies have shown a high percentage of misinformation on different health issues on social media [14,15]. Specifically regarding ADHD and TikTok, Yeung et al [16] have shown that content about ADHD on TikTok was predominantly misleading (52%), which includes inaccurate and overgeneralized information. Only 21% of the analyzed videos were considered useful, while the rest showed personal experiences [16]. With the increased “popularity” or “virality” of ADHD-related videos on TikTok in the last few years, and with the rapidly increasing number of videos on that topic, a repeated and updated analysis about misleading information tied to ADHD is needed.

Misleading information about ADHD on TikTok often includes incorrect descriptions of ADHD symptoms, the overpathologizing of behaviors that are common in the general population, and even “ADHD tests” that falsely claim to be able to provide diagnoses. Such misinformation can lead to misunderstandings about the disorder and even influence viewers’ behavior. While the health belief model is usually used to explain preventive health behavior, it can also be applied to understand the behavior of viewers of ADHD content on TikTok. The health belief model consists of 6 constructs: perceived susceptibility of the disease, perceived severity of the disease, perceived benefits, perceived barriers, self-efficacy, and cues to action [17]. Misleading content on ADHD, including the overpathologizing of behaviors that are common in the general population, may lead to the trivializing of the disorder, and viewers might perceive ADHD severity and burden of disease as lower. In addition, TikTok’s algorithm increases exposure to such videos, potentially increasing self-observance of certain own behaviors and therefore raising viewers’ perceived susceptibility to ADHD. Misinformation about ADHD and its diagnostic process, as well as personal experience reports and depictions of stigmatization toward ADHD, can affect viewers’ perceptions of the barriers and benefits of seeking a formal diagnosis and potentially lead to inappropriate health behaviors like self-identifying with ADHD symptoms and even self-medicating, as a study has shown that for mental illness, a high prevalence of undiagnosed individuals was self-medicating [18]. While only a medical professional can diagnose ADHD, this self-identification with symptoms and behaviors typical of ADHD is often referred to as “self-diagnosing” and is used accordingly in this study [12,19]. This self-identifying behavior can result in stress in people who perceive themselves as affected, which can cause comorbidities to go unrecognized and therefore delay receiving appropriate treatment and can even increase stigmatization by minimizing the effects that ADHD can have on affected people [12,20,21]. However, the effect of TikTok videos about ADHD on viewers has not yet been studied, and it remains unclear how often recipients identify with the ADHD information shared on TikTok and if, or how often, they may even self-diagnose.

The objectives of this study are (1) to analyze the quality and usefulness of the most popular videos about ADHD on TikTok and (2) to explore how content about ADHD is perceived by conducting an in-depth analysis of the viewers’ comments to these ADHD-related TikTok videos.

## Methods

### Ethical Considerations

This research did not involve the use of clinical data, human participants, or animals. All data analyzed were obtained from publicly available TikTok videos and creator accounts, and the research was conducted in accordance with the European General Data Protection Regulation. Creators’ and users’ names and other personally identifiable information were anonymized. As there was no direct participation of patients, no interaction with users, and no expected negative effects on creators, an ethical review was not deemed necessary, as per the RatSWD recommendations on research ethics self-assessment [22].

### Part 1: Video Analysis

#### Video Inclusion and Search Strategy

The methods used for the video analysis were based on Yeung et al [16]. They conducted their search for 100 videos on July 18, 2021 and in accordance with this, we aimed at including at least 100 videos on ADHD uploaded after July 18, 2021, in our analysis. Our inclusion criteria were videos that addressed ADHD and contained the word “adhd” in either the title or the hashtags. We excluded videos uploaded before July 19, 2021, videos other than the English language, videos that did not include audio or written text, videos that had the comment function disabled, videos that contained advertisements for products or services, or videos that primarily dealt with other diseases or comorbidities instead of ADHD.

We searched the TikTok mobile app on November 28, 2023, and November 29, 2023, with the hashtag “#adhd” for videos about ADHD. The results were sorted for likes count, with the most popular videos (the videos receiving the most “likes”) displayed at the top of the search.

#### Data Extraction

We downloaded or screen-recorded the 130 most-liked videos and extracted video data automatically using a commercial scraping software (“clockworks/free-tiktok-scraper,” provided by Apify) [23]. Extracted metadata on the video creators included the username of the creator, follower count, creator account type (private or public account, verified account, and business account), how many accounts the creator follows, origin of the creator, and the creator’s biography. Furthermore, metadata on the videos included the weblink of the video, number of likes or views or shares or saves or comments, time of video upload, hashtags, title, duration, and country of origin.

Business accounts are public profiles for brands, companies, or anyone who wants to promote their business (products or services) and use TikTok’s marketing tools. Verified accounts are accounts with a blue checkmark, which helps distinguish if the creator’s identity was confirmed and it is not a copycat, fan account, etc, indicating authenticity and credibility.

All videos were assessed for inclusion according to the aforementioned inclusion criteria.

#### Rating of Usefulness

Following the methodology of Yeung et al [16], all included videos’ contents were categorized as either “misleading,” “personal experience,” or “useful.” Videos were considered misleading if they contained false information on ADHD and if they showed symptoms not considered typical for ADHD (based on the German S3 guideline on ADHD and the *International Classification of Diseases, 11th Revision*) [24,25]. They were also considered misleading if they were overgeneralizing, for example, stating that “everybody with this symptom or behavior has ADHD,” or “everybody with ADHD has this symptom or behaves like this.” The category “misleading” therefore included, for example, videos claiming to provide a diagnostic test for ADHD, misleading points of view with wrong depictions of symptoms, and overgeneralizing statements. We considered videos “personal experience” if they described the creator’s personal experience with ADHD without being overgeneralizing, and videos as “useful” if they contained correct information on ADHD or correctly depicted symptoms and behaviors.

Videos could only be assigned to one of the three categories. Videos that depicted a personal experience but also contained misleading statements or useful information were categorized as misleading (or useful) rather than as personal experience. Videos, therefore, could only be categorized as personal experience if they were neither misleading nor useful.

Three members of the interdisciplinary research group independently rated the usefulness of the videos, using the German S3 guideline on ADHD and the *International Classification of Diseases, 11th Revision* [24,25]. Any disagreements were resolved through discussion. The research group comprised a physician, a clinical psychologist, and researchers in health science.

#### Quality Assessment

We assessed the informational quality of the videos using the Patient Education Materials Assessment Tool for Audiovisual Materials (PEMAT-A/V), a systematic method to assess the understandability and actionability of audiovisual patient education materials. Its goal is not to rate the quality of the information provided, but to assess if patients or users will be able to understand and act on the provided information. The PEMAT-A/V consists of 13 items assessing understandability and 4 items assessing actionability [26]. The items are scored as “agree=1 point” or “disagree=0 points” (and some items additionally have the option to choose “not applicable”). Final understandability and actionability scores are calculated by summing up the total achieved points and dividing them by the total possible points. The final score is reported as a percentage of the total possible points. The PEMAT-A/V tool was selected because it is well-suited for evaluating audiovisual health information and not only assesses understandability but also actionability. However, 3 of the 17 items cannot be rated if the material or video is shorter than 60 seconds, which is a common occurrence with TikTok videos.

Quality assessment was conducted by 2 members of the research group independently, and disagreement was resolved by discussion.

### Part 2: Comment Analysis

#### Data Extraction

By purposive sampling, we selected 6 videos, including 2 misleading videos, 2 useful videos, and 2 videos categorized as personal experience. We selected the videos based on the highest likes count in each category but made sure not to include 2 videos from 1 creator. We scraped a minimum of 900 first-level comments of these videos using a commercial scraping software (“clockworks/tiktok-comments-scraper,” provided by Apify) [23]. From the retrieved comments, a random sample of 100 comments per video was drawn using SPSS (IBM Corp). Scraped data on the comments included the comment text, the username of the person commenting, the time the comment was made, and the number of likes the comment received. We translated comments that were written in languages other than English, French, Spanish, Italian, or German using DeepL Translator (DeepL SE).

#### Content Analysis

We analyzed the content of user comments to investigate how often self-identification with ADHD-related behavioral patterns and self-diagnosis of ADHD among viewers occurred. The comments were categorized into 16 inductively derived categories, including 10 distinct categories directly regarding ADHD (1-10) and 6 rather technical categories (11-16). These 16 categories are explained in Table 1.

**Table 1.** Comment categories.

No.	Comment category	Explanation
1	Self-attribution of behavioral patterns	The person commenting attributes the shown behavioral patterns to themselves.
2	Self-attribution of ADHD^a^	The person commenting attributes ADHD to themselves.
3	Attribution of ‘ADHD typical behavioral patterns’ to others	The person commenting attributes the shown behavioral patterns to another person.
4	The attribution of ADHD to others	The person commenting attributes ADHD to another person.
5	“Behaviors don’t apply”	The person commenting does not relate to the shown behavioral patterns.
6	“I personally suffer from ADHD”	The person commenting states that they have ADHD.
7	“I personally do not suffer from ADHD”	The person commenting states that they do not have ADHD.
8	Questions on ADHD	The person commenting asks a question on ADHD topics.
9	Personal experience	The person commenting shares a personal story.
10	Shown behaviors are not typical for ADHD	The person commenting states that the shown behavioral patterns are not specific to people with ADHD, but that everybody behaves like that.
11	Comments on video content	The person comments on an aspect of the video that is not related to ADHD.
12	Tagging of other users	The person commenting tags another user.
13	Jokes or irony	The person commenting makes a joke or uses irony.
14	Others	Comments that do not fit the other categories.
15	Not interpretable	The comment can either not be translated appropriately into English or the true meaning of the comment cannot be understood.
16	Empty comments	Comments that were empty (no text, emojis, etc). The mechanism behind the scraping process did not explain why comments appeared empty.

a ADHD: attention-deficit/hyperactivity disorder.

Multiple categories could be ascribed to 1 single comment; for instance, a comment might both tag another user and include self-attribution of behavioral patterns (eg,*“@Kate this is me”*). However, a comment cannot self-attribute behavioral patterns and additionally say that behaviors do not apply, and since the use of irony alters the meaning, for our analysis, a comment also cannot self-attribute behavioral patterns of ADHD and use irony. Similarly, a comment could not simultaneously assert that the commenter both suffers from and does not suffer from ADHD.

Two members of the research group conducted the comment categorization independently, and disagreement was resolved by a third researcher and by discussion in the research group.

#### Statistical Analysis

For both parts of this study (part 1: video analysis and part 2: comment analysis), we calculated the interrater reliability and descriptive statistics.

Fleiss κ was used to calculate the interrater reliability between the 3 raters for the video categorization, and Cohen κ to calculate the interrater reliability between the 2 raters each for the PEMAT-A/V assessment and the comment categorization. We consider a κ statistic of >0.4 as moderate agreement and a κ statistic of >0.6 as substantial agreement between the raters [27].

To compare video characteristics and PEMAT-A/V scores between the useful, misleading, and personal experience videos, we used one-way ANOVA. For data with variance heterogeneity, the Welch ANOVA was used. We conducted post hoc analyses using the Games-Howell post hoc analysis. To compare the comments between useful, misleading, and personal experience videos, we used chi-square tests of independence. We considered a *P* value of *P*<.05 as significant for all analyses.

Statistical analyses were performed using SPSS (version 27).

## Results

**Figure 1.**
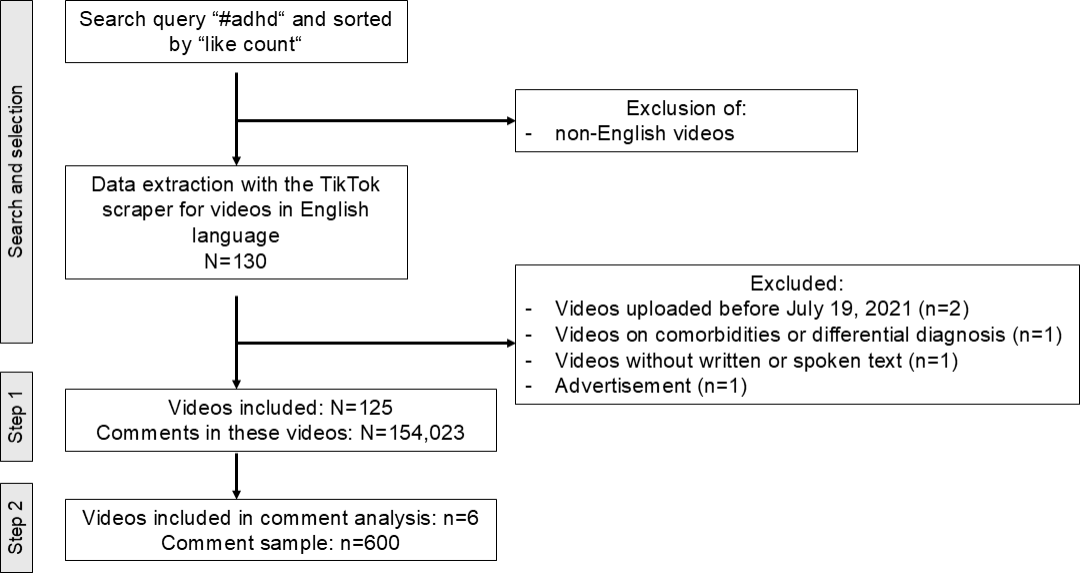
Flowchart of video and comment inclusion.

A total of 130 videos were scraped for information and after assessing inclusion and exclusion criteria, 125 videos were included in the final analysis, and 6 videos were selected and included in the comment analysis. The methodological approach of the video search and selection is illustrated in the flowchart in Figure 1.

### Part 1: Video Analysis

#### Video and Creator Characteristics

The 125 included videos were uploaded by 73 different creators from 13 different countries. Most videos were created in English-speaking countries (105/125, 84%) with the highest percentage in the United States (48/125, 38.4%). Most creators only had 1 video in the included 125 most-liked videos (54/73, 74%), but on average, creators had 1.7 (SD 1.54; range: 1‐8) videos in the most liked videos.

The videos had a mean duration of 44 seconds (SD 60 s), with the shortest video only lasting 7 seconds and the longest video 9 minutes and 47 seconds. On average, the videos had a total of 10.16 million views (SD 9.75 million) and 1.21 million likes (SD 1.16 million).

For more information on the creators and videos, see Table 2 and column “Total” in Table 3.

**Table 2.** Characteristics of creators (n=73) and videos (n=125).

Characteristics	Value
Creator	
English-speaking countries of origin^a^, n (%)	51 (70)
Verified account, n (%)	7 (10)
Business account, n (%)	3 (4)
Health care professional (self-proclaimed), n (%)	3 (4)
ADHD^b^-or life-coach (self-proclaimed), n (%)	6 (8)
Followers, mean (SD)	931,528 (2,002,991)
Uploaded videos, mean (SD)	626 (730)
Video	
Views, mean (SD)	10,156,800 (9,748,154)
Likes, mean (SD)	1,209,286 (1,156,636)
Saves, mean (SD)	112,520 (94,453)
Shares, mean (SD)	29,926 (31,306)
Comments, mean (SD)	13,958 (23,723)
Duration (s), mean (SD)	44 (60)
English-speaking countries of origin^c^, n (%)	105 (84)

a Other countries: Cyprus, Germany, Israel, Japan, Morocco, the Netherlands, and Poland.

b ADHD: attention-deficit/hyperactivity disorder.

c Other countries: Poland, Japan, Morocco, the Netherlands, Germany, Greece, Israel, Korea, and Malaysia.

**Table 3.** Video characteristics for all videos and divided into misleading, useful, and personal experience videos (n=125).

Characteristics, mean (SD; range; 95% CI)	Total	Misleading (n=63)	Personal experience (n=38)	Useful (n=24)	*P* value
Likes (in million)	1.21 (1.16; 0.35‐7.70; 1.00 to 1.41)	1.31 (1.35; 0.36‐7.70; 0.97 to 1.65)	1.28 (1.08; 0.35‐5.40; 0.93 to 1.64)	0.83 (0.50; 0.37‐2.50; 0.62 to 1.04)	.017
Views (in million)	10.16 (9.75; 1.70‐67.50; 8.43 to 11.88)	11.67 (11.71; 2.10‐67.50; 8.72 to 14.61)	9.92 (8.01; 1.90‐36.80; 7.28 to 12.55)	6.58 (4.47; 1.70‐23.20; 4.69 to 8.46)	.008
Saves (in thousand)	112.52 (94.45; 12.71‐580.71; 95.8 to 129.24)	128.09 (112.05; 25.08‐580.71; 99.87 to 156.31)	91.42 (76.16; 12.71‐363.60; 66.39 to 116.45)	105.06 (58.85; 28.56‐249.96; 80.21 to 129.91)	.156
Shares (in thousand)	29.93 (31.31; 1.33‐184.00; 24.38‐35.47)	27.68 (29.10; 2.13‐182.50; 20.35‐35.01)	33.41 (38.17; 4.54‐184.00; 20.86‐45.95)	30.31 (24.96; 1.33‐97.80; 19.77‐40.85)	.719
Comments (in thousand)	13.96 (23.72; 0.61‐214.00; 9.76 to 18.16)	19.55 (31.63; 1.67‐214.00; 11.58 to 27.52)	8.57 (9.12; 1.49‐44.10; 5.57 to 11.56)	7.82 (5.48; 0.61‐23.30; 5.50 to 10.13)	.022
Duration (s)	44 (60; 7‐587; 34 to 55)	35 (23; 7‐100; 29 to 40)	50 (92; 7‐587; 20 to 80)	59 (59; 10‐222; 34 to 84)	.106
Hashtags	6 (4; 0‐38; 5 to 6)	5 (3; 1‐18; 5 to 6)	6 (7; 0‐38; 4 to 8)	6 (3; 0‐11; 4 to 7)	.897
PEMAT-A/V^a^ actionability (%)	5.1 (18.0; 0‐100; 1.9 to 8.3)	5.3 (19.1; 0‐100; 0.5 to 10.1)	2.6 (9.1; 0‐33; –0.4 to 5.6)	8.3 (24.6; 0‐100; –2.0 to 18.7)	.415
PEMAT-A/V understandability (%)	79.5 (18.7; 29‐100; 76.2 to 82.8)	74.1 (20.3; 29‐100; 69.0 to 79.2)	80.5 (15.8; 33‐100; 75.3 to 85.7)	92.3 (10.4; 67‐100; 87.9 to 96.7)	<.001

a PEMAT-A/V: Patient Education Materials Assessment Tool for Audiovisual Materials.

#### Usefulness and Quality of Videos

Of the 125 included videos, 50.4% (63/125) of videos were rated misleading, 30.4% (38/125) showed a personal experience, and 19.2% (24/125) were useful. Agreement between raters was moderate, with a κ statistic of 0.529 (*P*<.001).

Useful videos often showed comorbid disorders that can occur alongside ADHD, such as auditory processing disorder, and explained relevant symptoms. Other useful videos explained relevant ADHD symptoms, such as impulsivity, difficulty focusing on and completing tasks, and hyperactivity. Personal experience videos mainly showed the creators’ point of view as individuals affected by ADHD. They showed the creators experiencing distraction while doing a task, procrastinating, being restless, experiencing self-doubt, and feelings of being overwhelmed. Misleading videos included videos that showed symptoms that can be related to ADHD but can also be “normal” behavior, such as being late or poor time management, fidgeting with random objects, and forgetfulness. Other misleading videos wrongly depicted anxiety as nervousness and as a symptom of ADHD, not a possible comorbidity; or creators explained “ADHD paralysis,” which they described as “my brain won’t let me do this task.” Some videos also included so-called “ADHD tests,” suggesting the viewers have ADHD if they experience the mentioned symptoms.

The 125 videos score on average 79.5% (SD 18.7%) in the PEMAT-A/V understandability domain, and 5.1% (SD 18.0%) in the actionability domain. For 3 PEMAT-A/V items, the agreement between raters could not be calculated (items 12, 19, and 25). The raters were in complete agreement for these items, as according to the PEMAT-A/V guidelines, they should be marked as “not applicable” if the audiovisual material is a video and if no tables, graphs, and charts are used. For the items with a significant agreement (11/17), the mean agreement was moderate, with a mean κ statistic of 0.508 (SD 0.187).

Video characteristics and quality for useful, misleading, and personal experience videos are described in Table 3.

Useful videos had the highest PEMAT-A/V understandability score, with an average score of 92.3% (SD 10.4%; *P*<.001). Post hoc analysis revealed significantly lower PEMAT-A/V understandability scores of the misleading and personal experience videos compared to the useful videos (mean difference 18.2%, *P*<.001 for misleading and 12.0%, *P*=.002 for personal experience videos). There was no statistically significant difference between the personal experience and the misleading videos (mean difference 6.4%; *P=*.190), and in the PEMAT-A/V actionability scores for useful, misleading, and personal experience videos (*P=*.415; Table 3).

Misleading videos were more popular than useful videos, as they had significantly more likes, views, and comments than useful videos (mean difference 476,113 likes*, P*=.047; 5,091,667 views*, P=*.012; 11,733 comments*; P=*.016). They also had more comments than videos on personal experiences.

### Part 2: Comment Analysis

On average, comments were assigned to 1.4 categories each, leading to a total of 840 assigned categories. Comments were most often assigned to “self-attribution of behavioral patterns” (220/600, 36.7%), followed by “personal experience” (93/600, 15.5%), regarding the categories directly related to ADHD. Regarding the technical categories, commenting persons most frequently “tagged other users” (115/600, 19.2%). Table 4 shows an overview of the frequency of all categories.

**Table 4.** Frequency of comments for all videos and divided into misleading, useful, and personal experience videos (n=600).

Comment category	Total, n (%)	Useful (n=200), n (%)	Misleading (n=200), n (%)	Personal experience (n=200), n (%)	*P* value
Self-diagnosis	252 (42)	79 (39.5)	64 (32)	109 (54.5)	<.001
Self-attribution of behavioral patterns	220 (36.7)	67 (33.5)	51 (25.5)	102 (51)	<.001
Self-attribution of ADHD^a^	32 (5.3)	12 (6)	13 (6.5)	7 (3.5)	.359
Attribution of “ADHD typical behavioral patterns” to others	9 (1.5)	2 (1)	0 (0)	7 (3.5)	.012
The attribution of ADHD to others	1 (0.2)	1 (0.5)	0 (0)	0 (0)	.367
“Behaviors don’t apply”	8 (1.3)	5 (2.5)	3 (1.5)	0 (0)	.090
“I personally suffer from ADHD”	28 (4.7%)	14 (7%)	5 (2.5%)	9 (4.5%)	.102
“I personally do not suffer from ADHD”	25 (4.2%)	9 (4.5%)	5 (2.5%)	11 (5.5%)	.311
Questions on ADHD	15 (2.5%)	10 (5%)	3 (1.5%)	2 (1%)	.020
Personal experience	93 (15.5%)	51 (25.5%)	15 (7.5%)	27 (13.5%)	<.001
Shown behaviors are not typical for ADHD	58 (9.7%)	14 (7%)	35 (17.5%)	9 (4.5%)	<.001
Comments on video content	71 (11.8%)	14 (7%)	35 (17.5%)	22 (11%)	.005
Tagging of other users	115 (19.2%)	33 (16.5%)	40 (20%)	42 (21%)	.486
Jokes or irony	53 (8.8%)	27 (13.5%)	20 (10%)	6 (3%)	<.001
Others	23 (3.8%)	7 (3.5%)	5 (2.5%)	11 (5.5%)	.282
Not interpretable	27 (4.5%)	8 (4%)	17 (8.5%)	2 (1%)	.001
Empty comments	8 (1.3%)	3 (1.5%)	0	5 (2.5%)	.09

a ADHD: attention-deficit/hyperactivity disorder.

To assess the prevalence of viewers “self-diagnosing” in the comments, we looked further into the categories “self-attribution of behavioral patterns” and “self-attribution of ADHD.” Agreement between raters for the category “self-attribution of behavioral patterns” was substantial, with a κ statistic of 0.638 (*P*<.001) and agreement for the category “self-attribution of ADHD” was moderate, with a κ statistic of 0.544 (*P*<.001). Examples of comments assigned to these categories include “wait so ur saying I might have adhd?” “Does that mean I have ADHD cause I do that,” and “me 100% of the time,*”* showing a varying degree of self-identification with the shown behavior or the disorder. Of the 600 comments, 220 (36.7%) comments solely indicated a self-attribution of ADHD-related behavioral patterns, and 32 of the 600 (5.3%) comments, a self-attribution of ADHD. In combination, 252 of the 600 (42%) comments indicate some sort of “self-diagnosis” with ADHD, which is shown in Table 3 under the newly summarized comment category “self-diagnosis.”

The number of comments indicating a possible self-diagnosis varied between misleading, useful, and personal experience videos, and the statistical analysis showed a significant association between the usefulness of the videos and the number of comments indicating a possible self-diagnosis. The strength of association was small (*χ*^2^_2_=21.55; *P*<.001; V=0.190). Comments indicating a possible self-diagnosis were most common in videos categorized as personal experience (109/200, 54.5%), followed by useful (79/200, 39.5%) and misleading videos (64/200, 32%).

If differentiated between self-attribution of behavioral patterns and the self-attribution of ADHD, there was a significant association between the usefulness of the videos and the number of comments indicating a self-attribution of behavioral patterns. The strength of association is small (*χ*^2^_2_=29.3; *P*<.001; V=0.221). Comments indicating a self-attribution of behavioral patterns were most common in videos categorized as personal experience (102/200, 51%), followed by useful (67/200, 33.5%) and misleading videos (51/200, 25.5%). The association between usefulness and the number of comments indicating a self-attribution of ADHD was not statistically significant (*χ*^2^_2_=2.05; *P*=.359; V=0.058).

Besides the comments hinting towards a potential self-diagnosis, the frequency of other comments was also found to be associated with the usefulness of the videos. The attribution of behaviors typical for ADHD to others was also most common in personal experience videos (7/200, 3.5%), followed by useful and misleading videos (2/200, 1% and 0/200, 0% respectively; *χ*^2^_2_=8.799; *P*=.012; V=0.121). Questions on ADHD, comments with personal experiences, and joking or ironic comments were most common in useful videos (*χ*^2^_2_=7.79, *P*=.02, V=0.114; *χ*^2^_2_=25.654, *P*<.001, V=0.207; *χ*^2^_2_=14.198, *P*<.001, V=0.154, respectively; Table 3). Comments on how the shown behavior is not typical for ADHD, comments on the video content but unrelated to ADHD, as well as not interpretable comments were most common in misleading videos (*χ*^2^_2_=21.797, *P*<.001, V=0.191; *χ*^2^_2_=10.767, *P*=.005, V=0.134; *χ*^2^_2_=13.264, *P*=.001, V=0.149, respectively; Table 4).

## Discussion

### Principal Results

Many users turn to TikTok for health information or even health advice [8,28]. However, many studies have shown that health-related information on social media, particularly TikTok, is often inaccurate or misleading [14-16]. Given that the number of TikTok videos on ADHD has increased substantially over the recent years, we have analyzed the most popular TikTok videos on ADHD regarding their usefulness, understandability, and actionability as health information. In addition, we have explored viewer perceptions of these videos by analyzing the comments.

The most popular videos on ADHD between July 2021 and November 2023 averaged 10.2 million views, which is a considerable increase from the 2.8 million views that Yeung et al [16] reported in their study in 2022. This suggests that TikTok videos on ADHD are trending more than ever. We found a high proportion of these ADHD-related TikTok videos to be misleading (63/125, 50.4%), which is in line with the results of a study by Yeung et al [16] from 2022, where 52% of the included videos were misleading. While the prevalence of misleading information varies by health topic [14], many other studies report high rates of misinformation, for example, Aragon-Guevara et al [15] found 73% of videos providing informational content on autism to be either inaccurate or overgeneralizing.

This study shows that the information provided in TikTok videos on ADHD is easily understandable, as evidenced by high PEMAT-A/V understandability scores averaging 79.5%. While videos classified as useful scored significantly higher than misleading and personal experience videos, even misleading videos achieved an understandability score of 74%. Since the PEMAT-A/V does not assess the accuracy of the information, this indicates that potential misinformation was presented in an easily understandable way, possibly facilitating the distribution of false information. PEMAT-A/V actionability scores, however, were generally low. TikTok videos might provide information that is easy to understand, but rarely give recommendations or describe specific actions for the viewers to take, for example,“see your health care provider” or “take this medication.” Yeung et al [16] also reported low actionability scores, although their scores were higher than we found in this analysis. In particular, the useful videos in the study by Yeung et al [16] showed higher actionability scores (27.8%), which could be because a higher percentage of useful videos in their study was created by health care professionals (7/21, 33.3%), who might be more inclined to recommend appropriate action compared to private individuals. In comparison, only one of the useful videos in our analysis was created by a health care professional (1/24, 4.2%).

The low actionability of ADHD videos on TikTok raises concerns. Viewers are constantly provided with easily understandable information on symptoms and might recognize those symptoms in themselves. But the lack of clear guidance or actionable steps—such as encouragement to consult a health care professional or to seek a professional diagnosis and guidance on how to do so—may lead to insecurity and act as a barrier to a formal diagnosis. Instead, viewers might self-diagnose and potentially also self-treat [18]. This is especially problematic for viewers with low health literacy. Clear guidance on subsequent actions, such as seeking a diagnosis and management of symptoms, would be highly beneficial, as these audiences might have difficulties accessing other health information and resources.

Our study found that misleading videos were the most popular, receiving the highest engagement through likes, views, and comments. However, the difference in likes and views between misleading videos and videos on personal experience was not significant. This is surprising, as other studies found that first-person and personal experience videos receive the most engagement, suggesting that social media users are drawn to relatable videos created by individuals who are personally affected by ADHD [16,29]. However, videos on personal experience that were misleading or overgeneralizing were categorized as misleading. Consequently, a substantial number of misleading videos also showed personal experiences, which might be one of the reasons why misleading videos in our analysis showed the highest engagement rates. The popularity of misleading videos is concerning. It has been shown that videos with high engagement are more likely to be further promoted by TikTok’s algorithm [30]. They appear more frequently on people’s For You Page, which increases their reach, can lead to even higher engagement rates, and consequently means that the misleading content is being distributed to an even wider audience. In addition, the algorithm pushes this content especially to viewers who have shown a previous interest in the topic or have engaged with similar videos. Repeatedly being shown individuals affected by ADHD, as well as mainly wrong depictions of symptoms, might increase viewers’ perceived susceptibility to ADHD, and together with the perceived low severity of ADHD that the misleading videos suggest, this might potentially increase self-identification [17].

Our analysis shows that misleading videos not only received the most comments in general, but compared to useful and personal experience videos, they also received significantly more comments that stated that the behaviors depicted were common to everyone, not just people with ADHD. A substantial number of people, therefore, commented to criticize the video, or to identify the information in the video as misleading. It is encouraging that many viewers are skeptical or disagree with misleading videos, and not all viewers self-identify with ADHD behaviors. However, their engagement with the video increases the reach of the video nonetheless.

A US survey showed that the most common reasons for using TikTok for health-related information are to receive advice from other people with similar health conditions, to receive support, or to get information on their disorders. Communicating with physicians and health care professionals ranked rather low on this list of reasons [8], explaining why personal experience videos might be so popular and why videos from health care professionals might be underrepresented in our sample of the most popular videos.

Since TikTok is primarily used for entertainment, it is not surprising that the explorative comment analysis revealed some comments as either joking or (supposedly) ironic in meaning (53/600, 8.8%). Many comments were also tagging other users (115/600, 19.2%), which can be understood as a form of online communication. Tagging of users is one way of sharing the video and many viewers were communicating with friends or acquaintances in the comments to the videos. Without further information, the interpretation of these comments is difficult, as the meaning of tagging another user can vary. Apart from sharing the video, it can also be interpreted as “that’s you” or even “that’s me,” or just to show that one might find this video interesting.

Viewers resonated with the behaviors depicted in the videos in a substantial number of comments, particularly in the personal experience videos. These videos, including “point-of-view” videos, show people with ADHD in everyday situations. Many viewers attributed these allegedly ADHD-typical shown behaviors to themselves, by commenting with phrases like “I’m so her,” “I do this all the time,” or “It’s like watching myself.” However, while comments relating to the depicted behaviors were most prevalent in personal experience videos, they appeared in the useful and misleading videos as well. Regarding the actual self-attribution of ADHD in the comments (eg, “I think I have ADHD,” “I really need to get evaluated,” or “Ok I counted and this is the 12th sign that I have ADHD”), the comments were less frequent and there was no significant difference between useful, misleading, and personal experience videos. These comments about relating to the behavior labeled as typical for ADHD in the video do not necessarily indicate an actual belief in having ADHD. As TikTok is a platform mainly used for entertainment, not all comments can be taken seriously. However, if many people who are not affected by ADHD relate to the shown behaviors, this can lead to an oversimplification of the disorder, potentially increasing stigmatization of affected people and reinforcing existing stereotypes rather than improving mental health understanding [12,20,21].

False assumptions that people have ADHD might lead more people to seek a professional diagnosis, potentially resulting in longer waiting times for diagnosis and an increased risk of overdiagnosis [31]. On the other hand, some individuals might just accept their self-diagnosis and therefore not seek a professional diagnosis, risking that comorbidities and differential diagnoses could be overlooked. In addition, if people believe that they are suffering from a disorder without getting the proper care and treatment, this can cause significant stress and anxiety [20,21]. Some individuals might even try to treat themselves without consulting a health care professional, possibly with inappropriate treatment options and medication.

TikTok is a platform where everyone can share videos and where the information in these videos is not fact-checked. Users, therefore, need to be cautious when watching videos on health-related topics and critically assess the information provided. Media and health literacy are needed for this, especially for children and teenagers, as they are the main users of TikTok. Even if TikTok users do not intentionally search for health information on TikTok, the probability of unintentionally being exposed to health information is high [8]. Users also underestimate their own susceptibility to misinformation, which poses a higher risk of following false health information, both consciously and subconsciously [8]. Furthermore, TikTok’s algorithm is designed to promote content based on the viewer’s previous interactions with similar content, as well as content with a high engagement rate, as is the case for misleading videos on ADHD [30]. Continuous exposure to misleading content that focuses on certain diseases or disorders can lead to distorted perceptions of the viewer’s own health and can worsen their mental health, especially for more impressionable viewers such as children and teenagers. The low actionability of these videos, which depict disorders without offering guidance or coping strategies, can further exacerbate these issues.

To counteract the vast amount of misleading information currently available on social media, it could be helpful if more health care professionals and educators step up and upload videos on health-related topics. These videos should be engaging, as we see that videos by health professionals are considered more boring, often lacking the entertainment value, trending sounds, and other creative and engaging strategies that other creators use [28]. By providing reliable resources, warning about the risk of, and debunking misinformation, health care professionals and organizations could become trustworthy information sources for TikTok users seeking health information. Although TikTok itself does not fact-check content, it does display a warning at the top of the search results indicating that TikTok is not a substitute for medical advice. However, this warning is only visible through the search function and not on videos that appear directly on the For You Page. Expanding these warnings to appear with all health-related videos could encourage viewers to critically assess the information and make more informed health decisions. In addition, it is important for health professionals to address social media–driven misconceptions and correct misinformation that potentially originate from social media during patient interactions.

### Limitations

We used the PEMAT-A/V to assess the informational quality of the videos. This tool can be used to assess the understandability and actionability of audiovisual patient education material. However, the TikTok videos included in this analysis were on average 44 seconds long, and 3 of the 13 understandability domains of the PEMAT-A/V require videos to be at least 60 seconds long to be rated. The PEMAT-A/V provides an overview of the quality of the included videos as informational or educational material; however, it is generally not ideally suited for such short videos, whose intended goal is more towards entertainment than education. However, the PEMAT-A/V does not only assess understandability but also actionability of the health information and is considered one of the better online health assessment tools [32].

Due to the purposive sampling strategy used for the comment analysis, generalization of the results to other videos on ADHD on TikTok is not possible. The videos included in the comment analysis were selected based on the categories of usefulness assigned beforehand and the analyzed engagement rate of the videos, but other videos could have yielded different results and therefore would have potentially led to different conclusions regarding the self-identification of viewers with the disorder.

In addition, the categorization of the comments on TikTok proved difficult. Viewers have diverse cultural and linguistic backgrounds; they are nonnative English speakers or post comments in other languages; abbreviate words or phrases; and use humor, irony, emojis, and slang. These factors, together with the varied professional backgrounds, ages, and social media familiarity of the researchers, led to differing interpretations of some comments, necessitating discussions to reach a consensus. TikTok is primarily a platform used for entertainment, and while we assessed humor and irony and subsequently excluded those comments, it remains unsure how many commenters really are serious about their self-diagnosis. It is possible that despite the extensive discussion between the 5 different researchers and efforts to ensure interrater reliability, some comments were misunderstood and miscategorized, and that the number of viewers potentially “self-diagnosing” therefore differs. Nevertheless, the analysis gives a valuable first impression of how TikTok users perceive and engage with videos that, to a great extent, contain false and misleading information.

### Conclusions

ADHD is a popular topic on TikTok and videos on the topic are viewed by millions of users. While social media and TikTok have the potential to be an easily accessible and free source of health information for the population, the high prevalence of misleading and overgeneralizing content related to ADHD on TikTok is concerning. The widespread dissemination of this easily understandable information through the TikTok algorithm, together with the lack of actionable guidance in the videos, presents the risk of false self-identification and misdiagnosis with ADHD and can cause unnecessary stress, delayed appropriate treatment, unrecognized comorbidities, and increased stigmatization. It is important for viewers to critically evaluate information on health-related topics on social media and to assess the source or creator of this information. For health care professionals, it is important to address misconceptions arising from such platforms, and it would be beneficial for them to also use TikTok and produce engaging and accurate content to counterbalance the prevalent misinformation.

## Abbreviations

ADHD: attention-deficit/hyperactivity disorder
PEMAT-A/V: Patient Education Materials Assessment Tool for Audiovisual Materials
